# Study protocol of the sweet tooth study, randomized controlled trial with partial food provision on the effect of low, regular and high dietary sweetness exposure on sweetness preferences in Dutch adults

**DOI:** 10.1186/s12889-022-14946-4

**Published:** 2023-01-11

**Authors:** Eva M. Čad, Claudia S. Tang, Hanne B. T. de Jong, Monica Mars, Katherine M. Appleton, Kees de Graaf

**Affiliations:** 1 Division of Human Nutrition and Health, Wageningen University, Wageningen, The Netherlands; 2 Department of Psychology, Faculty of Science and Technology, Bournemouth University, Bournemouth, UK

**Keywords:** Sweetness, Sweet taste, Food preferences, Exposure, Taste perception

## Abstract

**Background:**

Several health organizations recommend lowering the consumption of sweet-tasting foods. The rationale behind this recommendation is that a lower exposure to sweet foods may reduce preferences for sweet tasting foods, thus lowering sugar and energy intake, and in turn aiding in obesity prevention. However, empirical data supporting this narrative are lacking. In fact, relatively little is known about the contribution of long-term sweet taste exposure on one’s sweetness preferences.

**Methods:**

The primary objective of this randomized controlled trial is to assess the effect of low, regular and high dietary sweetness exposure on preference for sweet foods and beverages, and to compare these effects between intervention groups. One hundred and eighty adults aged 18–65 years with a BMI of 18.5–30.0 kg/m^2^ will be recruited and randomly allocated to either: low dietary sweetness exposure (LSE) (10–15% daily energy from sweet tasting foods), regular dietary sweetness exposure (RSE) (25–30% daily energy from sweet tasting foods), or high dietary sweetness exposure (HSE) (40–45% daily energy from sweet tasting foods), for 6 months, followed by a 4-month follow up. Intervention foods are provided ad libitum, covering approximately 50% of the daily number of food items, to include sugar-sweetened, low-calorie-sweetener-sweetened and non-sweet foods. The primary outcome measure is the difference in change in sweetness preference from baseline to 6 months between intervention groups. Secondary outcomes include: change in sweet taste preferences at different time-points; taste intensity perception; behavioral outcomes: food choice and intake, sweet-liker type, food cravings, dietary taste preferences and dietary taste patterns; anthropometric outcomes: body composition, waist-hip circumference, body weight; and biochemical outcomes: glucose variability and biomarkers related to CVD and diabetes.

**Discussion:**

This study will generate important data on the effect of dietary sweetness exposure on sweetness preferences in terms of effect size and change, duration of change and its impact on food intake, body weight status and associated health outcomes.

**Trial Registration:**

The study protocol has been registered on ClinicalTrials.gov (ID no. NCT04497974, Registered 4 August 2020, https://clinicaltrials.gov/ct2/show/NCT04497974) and approved by Wageningen’s Medical Ethical Committee (ABR no. NL72134).

## Background

Sweetness is induced by sugars (mono and disaccharides) and other nutritive and non-nutritive agents which account for a considerable amount of energy intake [1]. Although the consumption of dietary sugars has changed since the early diets of our predecessors, in recent years (last decade), the intake of sugars has remained stable in some countries, and has decreased in others, such as in the Netherlands [2, 3]. Dietary guidelines advise limiting the consumption of free sugars in the diet to < 10% of total energy intake [4, 5]. Additionally, several public health organizations, such as the World Health Organization (WHO), advise reducing the consumption of sweet-tasting foods and beverages [6–8]. These recommendations are based on the assumption that a reduction in dietary exposure to sweet taste, regardless of the source of the sweet taste, will help people adapt to lower sweetness levels, thereby supporting adaptation to lower sugar and energy intake, and contributing to obesity prevention [9]. Although this narrative is simple and attractive, empirical data supporting it are lacking.

Sweet taste is innately liked [10]. Little is known about how and to what extent the innate preference for sweet taste can be modified by experiences later in life [2, 9]. Preferences for sweet taste intensities vary greatly between individuals, of which a part has a genetic basis [2, 9, 11, 12]. For salt, several studies have suggested that preferences can shift in humans in response to long-term changes in dietary salt levels [13, 14]. However, unlike the case with salt, the current evidence suggests that long-term exposure to sweet tasting foods or beverages may affect individuals’ sweetness intensity perception, but not their preferences [15]. Wise et al. (2016) [15] showed that when people are exposed to a low-sugar diet for three months they perceive a given concentration of sucrose in food and beverages to be more intensely sweet than it was before the diet, yet their optimal sweetness preference levels in food and beverages remained unchanged [15]. A recent review by Appleton et al. (2018) [16], summarising 21 long-term, short-term and cohort studies, cautiously concludes that in the short term (< 1 month) sweetness exposure may reduce sweetness preferences, however data from longer-term studies were limited and equivocal.

The mechanisms that may underlie changes in sweet taste, or sugar, intakes in response to sweet taste exposure remain unknown. The ‘common knowledge’ hypothesis, as above, proposes that exposure affects liking or preferences which then affect intakes. Some evidence suggests that long term exposure can affect sweet taste intensity perception, and these changes may occur through adaptations in taste receptors or changes in signal transduction pathways [15, 17, 18]. Such adaptations may also impact sweetness liking or preferences, or may result in sequential changes in intakes as a result of habituation, tolerance, cravings, or diminished reward sensitivity [19]. Thus, changing the taste of a diet could affect food liking, wanting and cravings, alongside sweetness perception. The evidence available points most strongly to changes in liking, preferences or pleasantness [16]. However, while some studies report decreases [20] or no change [21] in cravings for sweet foods after selective food deprivation, other studies report associations between cravings for sweet foods and intake of these foods [22–24]. Though the directionality of these positive associations are unknown, it is possible that sweetness exposure might impact the desire for sweet foods. To our knowledge, there are no long-term studies conducted on the effect of sweet taste exposure, without caloric restriction, on food cravings. It may be argued that food cravings lead to (increased) consumption, however, it is equally probable that repetitive consumption of specific foods contributes to cravings for those foods [23]. Given the ‘common knowledge’ hypothesis and the greater evidence for changes in sweet taste preferences, our focus for the trial is on the impact of exposure on liking / preferences. We will also measure sweetness perception, cravings and desire to eat as alternative mechanisms.

Studies assessing the link between sweetness exposure and sweetness preference often focus only on the manipulation of free sugars, specific sweet foods, or food groups, like beverages or high-energy dense snacks [25–28], instead of overall dietary sweetness exposure. So far, only two studies used an overall dietary sweetness exposure approach: a study by Wise et al. (2016) and a study by Griffioen-Roose et al. (2012) [15, 29]. The latter study investigated the effect of three different diets (sweet, savoury and mixed) on food preferences, and showed that a higher exposure to sweet foods resulted in a relatively lower liking, wanting and subsequent intake of sweet foods. However, the dietary intervention by Griffioen-Roose et al., (2012) lasted only 24-h and the findings may be attributed to sensory specific satiety. The intervention by Wise et al., (2016), lasted for three months, but in this study diets were defined based on total sugar intakes rather than dietary sweetness as a whole.

Preferences for sweet taste are often assessed in a single test food or beverage [30–36], even though it has been established that the preference for certain sweetness levels strongly depends on the food matrix [37, 38]. For instance, a sugar concentration of 10% (w/w) is optimal in soft-drinks, whereas a concentration of 30% (w/w) is found to be optimal in cakes [38]. Furthermore, it is well known that taste-taste interactions, flavour-taste interactions and sensations such as temperature, visual, olfactory and auditory stimuli may influence taste perception [24]. Likewise, the sweetener source, range of presented stimuli, and past experience with foods can affect individuals’ preference for sweetness concentration. It is probable that, sweetness preferences in familiar, commonly eaten foods, may be harder to change compared to those for unfamiliar foods, because there is no sweetness level associated with unfamiliar foods. Thus, preference shifts should be assessed across a variety of foods that differ in food matrix and familiarity, using different sources of sweetness which are distributed across a wide concentration range.

The relationship between exposure to sweet foods and body weight status is also poorly understood [9, 15]. Some observational studies do report an association between free sugar intake and body weight [see [39] for a review]. Additionally, there is strong evidence that the consumption of sugar sweetened beverages leads to weight gain [40, 41]. But more importantly, the scientific evidence linking the sweetness of an entire diet with energy intake, body weight and other health outcomes is weak at best [2]. It has been established that people with overweight and obesity have similar sweetness preferences compared to normal-weight individuals [9, 42–45]. In addition, it has been shown that sweetness in the diet is related to mono- and disaccharides present in the diet, but not necessarily energy content [26, 27, 46]. Although previous studies have reported that exposure to and liking of salty and fatty foods were positively related to body weight status, this is not the case for exposure to and liking of sweet foods [47–49]. There is a strong need to elucidate the effects of dietary sweetness exposure on weight status and other health related outcomes.

In summary, the link between long-term dietary sweetness exposure and preferences for sweet food is poorly understood. Hence, a long-term, sufficiently- powered study with a ‘whole diet’ approach is needed to investigate whether or not sweet taste preferences can be changed, either stimulated or suppressed, by variations in sweetness exposure. With this in mind, the Sweet Tooth study was designed. This study will provide evidence on the potential adaption of sweetness preferences, in terms of effect size and direction, duration and time-course of effects, and impacts on taste intensity perception, food choice and intake, sweet-liker type, food cravings, dietary taste preferences, dietary taste patterns, body composition, waist-hip circumference, body weight, glucose variability, and biomarkers related to CVD and diabetes. Given the variety of measurements to be completed, the purpose of this article is to describe the Sweet Tooth study protocol.

## Methods and design

### Objectives

Primary objective: To assess the effect of a 6-month low, regular and high dietary sweetness exposure on preference for sweet foods and beverages, and to compare these effects between the intervention groups. We hypothesise that regardless of the sweetness exposure level, preferences for sweet foods and beverages will not change from baseline to 6 months.

Secondary Objective(s): To assess the effect of a 6-month low, regular and high dietary sweetness exposure on taste intensity perception, behavioural outcomes: food choice and intake, sweet-liker type, food cravings, dietary taste preferences, dietary taste patterns; anthropometric outcomes: body composition, waist-hip circumference, body weight; and biochemical outcomes: glucose variability, and biomarkers related to CVD and diabetes.

### Study design

The Sweet Tooth study is a 6-month parallel randomized controlled trial with partial food provision. The study has been approved by the Wageningen Medical Ethical Committee (ABR nr. NL72134) and has been registered at ClinicalTrials.gov (ID no. NCT04497974). All procedures performed in this study are in accordance with the Declaration of Helsinki [50].

**Fig. 1:**
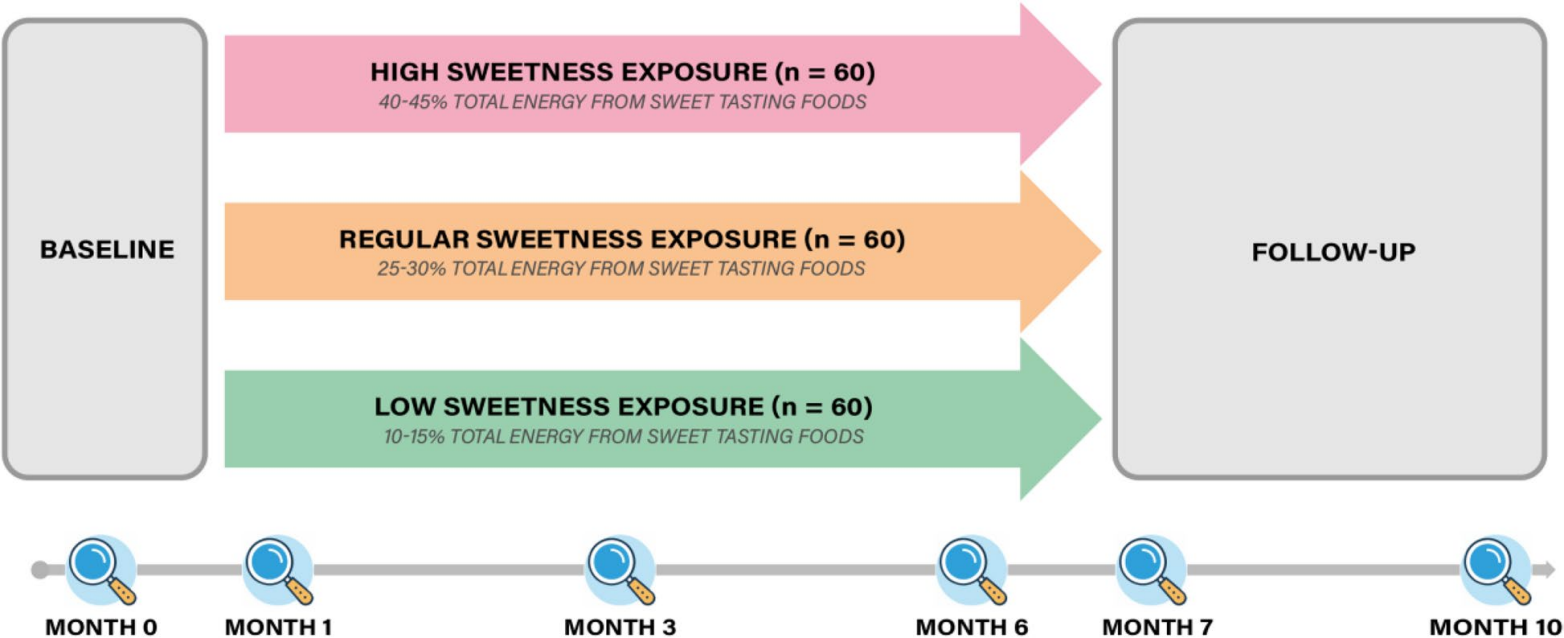
Overview of Sweet tooth study design, a RCT on the effect of 6-month low, regular and high dietary sweetness exposure on preference for sweet foods and beverages. Magnifying glass icons represent assessment visits conducted at baseline (month 0), 1, 3, 6, 7 and 10 months

An overview of the study design is shown in Fig. 1. The recruitment and entry phase will take approximately three years. Volunteers will be enrolled in the study in small groups throughout that time span, resulting in completion of the study at all times across the calendar year. Following a screening procedure, eligible participants are randomly allocated to either: a regular sweet exposure (RSE, control); a low sweetness exposure (LSE); or a high sweetness exposure (HSE) intervention. The intervention lasts for 6 months and the follow-up period is 4 months. The length of the sweetness exposure manipulation was chosen because the 3-month diet manipulation by Wise, Nattress [15] might not be sufficient to observe changes in preferences for sweet foods. Other studies however, investigated and reported changes to sweet food intakes over a 6-month period (see Appleton et al., 2018 [16]). These changes in intakes suggest that changes in preferences may occur over 6 months. Longer intervention periods might also result in more robust and longer-lasting effects on changes in sweetness intensity perception and food intake, than those demonstrated after 3 months, although concerns over adherence with longer study durations may also arise [51]. Concerns over the practical implications and likely adherence to a long intervention were also considered. The 1-month follow-up assessment was also based on the study by Wise et al., where changes to sweetness perception that were detected after 3 months reverted almost entirely within 1 month of the return to usual diets. An additional 3- month follow-up period to result in a 4-month follow-up in total was added to investigate changes over the longer term. Three months was chosen as half the length of the intervention and was again considered practical for participants and researchers.

The present study is monitored by BioFortis (https://www.merieuxnutrisciences.com). Monitoring involves an onsite visit(s) and online meetings. Monitoring of the present study ensures transparency, appropriate records of study procedures, ethical conduct, appropriate subject interaction and recruitment, and ensures high quality data collection and data management.

### Sample size estimation

The sample size calculation is based on our primary outcome: change in most preferred sweet taste concentration from 0–6 months. Previous studies have established that it is possible to detect shifts in preferred concentration from preference tests [36, 52]. For example, looking at the study of Liem and de Graaf [52] and the change in most preferred concentration, we can observe that mean ranking score for the sweet exposure group changed by around 0.4 and by around 0.6 for the sour exposure group from baseline to after exposure, which is around 10%. Therefore, the effect size of 0.1 was considered to be a relevant and meaningful effect size for our study, and was used to estimate the sample size. We estimate that 147 participants are needed to detect an effect size of 0.1, assuming a parallel study with 3 groups, with two repeated measures (baseline vs 6-month, correlation between measures of 0.7), and a power of 80% at a significance level of 0.05. To account for a potential dropout of 20%, 180 individuals will be enrolled in the study.

### Study participants

The Sweet Tooth study aims to include 180 participants aged 18 to 65 years. The study is conducted among individuals that are proficient in Dutch, are living in the Netherlands, and can visit Wageningen University and Research for all test sessions. Participants sign a written consent and are blinded to the real purpose of the study. Participants are told that the study aims to investigate the effect of the sensory properties of foods, like texture, taste and colour, on glucose metabolism and biomarkers related to risk for diabetes. The true aim of the study is revealed to the participants at the end of the study, when participants have completed the follow-up period. Participants are informed that their participation is completely voluntary and that they can drop out at any time without further explanation. They are compensated for their participation.

#### Eligibility criteria

Participants are eligible if they have normal weight or are overweight (BMI: 18.5—30 kg/m^2^), are aged between 18 and 65 years and have good general health. Participants are not eligible if they have abnormal blood glucose values (fasting glucose level: ≥ 6.1 mmol/L; non-fasting glucose level ≥ 7.8 mmol/L) or have established diabetes mellitus or insulin resistance, have endocrine, metabolic or other medical condition(s) that would influence glucose metabolism, have an eating disorder(s), taste or/and smell disorder, have gained or lost more than 3 kg in the last three months prior to study entry, suffer from lack of appetite, use medication that may influence taste perceptions and/or glucose metabolism, have a food allergy or/and food intolerances, are pregnant or lactating, have an excessive alcohol consumption (> 14 glasses/week), use soft or hard drugs, are a student or personnel of the division of Human Nutrition and Health at Wageningen University, or are participating or planning to participate in another study, for the duration of the study. Participants are withdrawn from the study in the case of pregnancy and in case of systematic weight change (gain or loss) of 4 kg or more over any 3-month period during the study intervention. Withdrawal by cause of systematic weight change was implemented due to ethical concerns.

#### Recruitment/screening

Participants are recruited via a pre-existing participants database (division of Human Nutrition and Health, Wageningen University), internet-based advertisements, printed media and flyer distribution. Participants who appear eligible from the questionnaire are invited for a screening appointment for a clinical assessment and taste tests to confirm eligibility. During this visit, weight and height are measured and participants are screened for normal blood glucose levels, using a finger prick (FreeStyle Freedom Lite, Abbott, UK) and for their ability to taste (total score: ≥ 12), using a validated, standardized Taste Strip Test developed by Mueller et al., (2003) [53]. Eligibility is judged by an independent medical investigator. Additionally, at the screening appointment the sweet liker phenotype [54, 55] is established via a liking test which is discussed in more detail below.

#### Randomisation, allocation concealment and sequence generation

Data collected at the screening visit are used to assign participants to intervention groups, based on sex (2 levels: male, female), age (3 levels: 18–34, 35–49, 50–65), Body Mass Index (BMI) (2 levels: 18.5–24.9, 25–30 kg/m2) and sweet liker phenotype (3 levels: sweet liker, inverted U, sweet disliker), in a process of stratified randomization (2 × 3x2 × 3 = 36 strata) to minimize differences between intervention groups in these baseline characteristics [56]. Treatment allocation is performed according to a computer-generated random schedule, at the ratio 1:1:1 to each of the three groups. The treatment allocation is performed by an independent person, not involved in the study outcome assessments or statistical analyses. The independent staff member maintains the randomisation list in a secure place with limited access to authorised personnel. The study is triple-blind – that is participants, researchers and analysts are blind to the treatment allocation during data collection and data analyses. Participants are asked not to disclose the foods they are consuming to the researchers. Researchers conducting outcome assessments and statistical analyses will remain blinded until the completion of all statistical analyses at group level.

### Dietary intervention

The Low Sweetness Exposure (LSE) group will be asked to consume foods to allow 10 – 15% total energy intake to come from sweet tasting foods. This is comparable to the Dutch Government recommendations [57] in which about 15% from the energy comes from sweet tasting foods [1]. The Regular Sweetness Exposure (RSE) group will be asked to consume a diet with 25 – 30% total energy intake from sweet tasting foods. This is similar to the average amount of sweet-tasting products consumed by the members of Dutch population, as described by van Langeveld et al. (2018); in this study it was observed that in the average Dutch diet about 28% of the energy consumed comes from sweet-tasting foods. Last, the HSE group will be asked to consume a diet with 40 – 45% energy from sweet tasting-foods. This proportion of sweet-tasting foods is also consumed, given free choice, by members of the Dutch population as demonstrated in the Dutch Food Consumption Survey 2007–2010 (DNFCS). The three intervention groups differ in exposure to sweet-tasting foods. The intervention diets of the three intervention groups are comparable in macronutrient composition, that is energy provided by fat, protein, carbohydrates and fibres. See Table 1 for the macronutrient composition of the three intervention diets, averaged across a 28-day rotating menu. The sweet taste of the diet comes from various sources, including natural sugars and non- and low-calorie sweeteners (LCSs). The interventions are based on a new methodology developed by van Langeveld et al. [1] which enables us to assess and quantify sweetness exposure within the Dutch diet, by profiling foods based on six taste clusters: Neutral; Salt, Umami & Fat; Sweet and Fat; Sweet and Sour; Fat; Bitter.

**Table 1:** Energy and macronutrient composition of the intervention diets (including intervention and other foods) per day averaged over the 28-day rotating menu. The diets were calculated using estimated energy needs of 2200 kcal, of an average adult woman

Nutrients	Low Sweet Exposure	Regular Sweet Exposure	High Sweet Exposure
Energy (MJ) (kcal)	9.2 (2,191)	9.3 (2,207)	9.3 (2,206)
Protein (g) (en%)	92.1 (17.0)	88.6 (16.3)	84.6 (15.5)
Fat total (g) (en%)	85.4 (34.4)	85.6 (34.2)	82.9 (33.1)
Saturated fat (g) (en%)	21.9 (9.0)	22.5 (9.2)	24.6 (10.0)
Carbohydrates (g) (en%)	244.4 (45.2)	251.1 (46.1)	260.5 (47.8)
Mono and disaccharides (g) (en%)	66.2 (12.1)	84.2 (15.3)	98.1 (17.8)
Polysaccharides (g) (en%)	175.7 (32.1)	163.6 (29.7)	160.4 (29.1)
Dietary fibre (g)	36.5	36.4	39.2
Sodium (mg)	2,375	2,031	1,984

During the 6-month intervention participants are provided with 50% of the food items from their allocated diet. Intervention foods are provided ad libitum, and participants can consume from these as they wish, thus a surplus of foods is provided without instructions on the amount of food that should be consumed. The provided intervention foods include sugar-sweetened, low-calorie-sweetener-sweetened and non-sweet foods. The average weight and macronutrient composition of provided foods for each intervention group is shown in Table 2. Additionally, Table 2 shows the total energy for two weeks' worth of sweet and non-sweet foods that are provided as intervention foods. The amount of energy from the provided foods is distributed as follows: 28,317 kcal for high sweetness exposure, 28,014 kcal for regular sweetness, and 27,799 kcal for low sweetness exposure which translates into approximately 2000 kcal per day, with provisions not being energy requirement-dependent. Participants are instructed to adhere to diet menu plans that include both provided intervention foods and other, non-provided foods like bread and vegetables. The provided intervention foods are intended to make up about 50% of the food items consumed. The actual intake of provided foods is monitored via dietary assessment methods (described in Compliance measures) and checklists.

**Table 2:** Macronutrient composition and weight of the provided intervention foods (sweet and non-sweet) per 2 weeks for each intervention group

		**Low Sweet Exposure**	**Regular Sweet Exposure**	**High Sweet Exposure**
Energy (MJ (kcal))	Liquids	13.1 (3,140)	14.1 (3,370)	14.5 (3,475)
	Semi-solids	40.9 (9,764)	40.7 (9,735)	40.9 (9,779)
	Solids	62.3 (14,895)	62.4 (14,909)	63.0 (15,063)
	**Total**	**116.3 (27,799)**	**117.2 (28,014)**	**118.5 (28,317)**
Protein (g)	Liquids	288.0	287.0	268.0
	Semi-solids	329.3	242.5	241.3
	Solids	475.8	232.6	253.0
	**Total**	**1,093.1**	**762.1**	**762.3**
Fat total (g)	Liquids	24.0	24.0	80.0
	Semi-solids	758.9	654.6	568.3
	Solids	789.0	719.1	661.9
	**Total**	**1,571.9**	**1,397.7**	**1310.2**
Carbohydrates (g)	Liquids	426.0	486.0	373.0
	Semi-solids	361.7	646.7	868.9
	Solids	1380.5	1766.9	1972.7
	**Total**	**2,168.2**	**2,899.6**	**3,214.5**
Mono and disaccharides (g)	Liquids	434.0	494.0	357.0
	Semi-solids	278.4	503.6	827.6
	Solids	827.6	948.1	1,474.0
	**Total**	**952.8**	**1,945.7**	**2,658.6**
Weight (g)	Liquids	14,000	14,500	14,500
	Semi-solids	4,465	4,015	3,820
	Solids	3,339	3,134	3,171
	**Total**	**21,304**	**21,149**	**21,491**

Participants receive monthly diet booklets which include daily menus (see Appendix 1 for daily menu examples), instructions on which foods to eat, checklists and useful recipes. Provided intervention foods are home-delivered on a biweekly basis. At the beginning of the intervention period, participants meet with a research dietitian to receive comprehensive dietary counselling and guidance. Moreover, throughout the intervention, participants regularly discuss their diet with the dietitian to ensure that the diet is clearly understood. Checklists are used to record the daily consumption of provided foods, thereby facilitating monitoring and dietary compliance. These checklists are checked by the research dietitian on a monthly basis.

Considering that consumption of liquid foods is associated with a lower taste exposure compared to solid foods [58, 59], particular emphasis has been given to the distribution of liquids, semi-solids and solids between the intervention groups (see Table 3). The exposure manipulation is achieved by supplying people with foods differing in taste profiles which are determined based on the Dutch taste database developed by Langeveld et al., 2018 [48]. Throughout the intervention all three groups receive different proportions of sugar-sweetened, low-calorie-sweetener-sweetened and non-sweet foods. To create a difference in sweetness exposure, the proportion of sweet-tasting foods vary group-wise: 7% of the foods provided for the LSE group are sweet tasting, 35% of the foods provided for the RSE group are sweet tasting and 80% of the foods provided for the HSE group are sweet-tasting. Among the LCSs present in the commercially available provided foods are saccharin, sucralose, steviol glycosides, maltitol, and isomalt. Previous research conducted in the Netherlands has shown that dietary taste patterns vary most during snacking and breakfast occasions [60]. For this reason, the provided intervention foods mainly include breakfast and snack items, such as bread toppings, dairy products, nuts, chocolates, crackers (see Appendix 2, for graphical representations of the provided intervention foods). To increase variety, participants also receive other sweet tasting products, not previously classified into taste clusters by Langeveld et al., 2018 [48] (5% of all provided foods). These foods include mostly bread toppings with added fruits, such as: hummus with mango, vegetables spread with pineapple, sweet onion chicken spread.

**Table 3:** Proportion of sweet tasting liquid, semi-solid and solid intervention foods provided per intervention group

		**Low Sweet Exposure**	**Regular Sweet Exposure**	**High Sweet Exposure**
**Liquids (#)**	Products containing nutritive sweetener^a^	0	1	0
	Products containing low & non-nutritive sweetener^a^	0	0	9
	**Total**	**0**	**1**	**9**
**Semi-solids (#)**	Products containing nutritive sweetener^a^	0	1	10
	Products containing low & non-nutritive sweetener^a^	0	3	3
	**Total**	**0**	**4**	**13**
**Solids (#)**	Products containing nutritive sweetener^a^	1	8	10
	Products containing low & non-nutritive sweetener^a^	2	1	1
	**Total**	**3**	**9**	**11**
	**Total number of provided sweet tasting foods (% of sweet tasting products provided)**	**3 (6.6%)**	**14 (35%)**	**33 (80%)**

^a^Number of sweet tasting intervention food items that are delivered to participants on a biweekly basis

### Study outcomes

Table 4 outlines the study outcome measurements and data collection methods to be used at each specified time-point. The primary outcome measure is change in sweet taste preference from baseline to 6 months. Secondary outcomes include change in sweet taste preference from baseline to 1, 3, 7 and 10 months; taste intensity perception; behavioral outcomes: food choice and intake, sweet-liker type, food cravings, dietary taste preferences and dietary taste patterns; anthropometric outcomes: body composition, waist-hip circumference, body weight and body height; biochemical outcomes: glucose variability, and biomarkers related to CVD and diabetes.

**Table 4:** Overview of Sweet Tooth outcome measures and data collection methods

Domain	Outcome to Be Measured	Data Collection Method	Baseline	Intervention			Follow-up	
				**1Month**	**3 Months**	**6 Months**	**7 Months**	**10 Months**
**Food taste preference**	Sweetness preferences	Rank-rating scale	✓	✓	✓	✓	✓	✓
	Saltiness preferences		✓	✓	✓	✓	✓	✓
**Taste intensity perception**	Sweet taste perception	100-unit VAS	✓	✓	✓	✓	✓	✓
	Salt taste perception		✓	✓	✓	✓	✓	✓
**Behavioural outcomes**	Food choice	Food choice from a buffet	✓	✓	✓	✓	✓	✓
	Food intake	Amount consumed from a buffet	✓	✓	✓	✓	✓	✓
	Sweet-liker type [54]	100-point VAS	✓	✓	✓	✓	✓	✓
	Taste preferences	PrefQuest^a^	✓	✓	✓	✓	✓	✓
	Food cravings	CoEQ	✓	✓	✓	✓	✓	✓
	Dietary taste patterns	Taste FFQ^b^	✓	✓	✓	✓	✓	✓
**Anthropometric outcomes**	Body composition	DEXA	✓			✓		✓
	Waist-hip circumference	Measuring tape	✓	✓	✓	✓	✓	✓
	Weight	Digital scale	✓	✓	✓	✓	✓	✓
**Biochemical outcomes**	Biomarkers related to CVD and diabetes	Fasting blood sample	✓	✓	✓	✓	✓	✓
	Glucose homeostasis	Glucose sensor^c^	✓			✓		✓
**Compliance**	Biomarkers of compliance	Urine sample (24-h sample)	✓	✓	✓	✓	✓	✓
	Dietary intake	Online 24-h recall	✓	✓	✓	✓	✓	✓
**Intervention Moderators**	Physical activity	SQUASH	✓	✓	✓	✓	✓	✓
	Adverse events, medication use	Questionnaires^b^, Study diet diary	✓	✓	✓	✓	✓	✓

*CoEQ* Control of eating questionnaire [61], *CVD* Cardiovascular disease, *DEXA* Dual-energy x-ray absorptiometry, *SQUASH* Short questionnaire to assess health enhancing physical activity [62], *VAS* Visual analogue scale

^a^Translated and Modified for the Dutch population based on Deglaire et al., 2012 [63]

^b^Developed for the Sweet tooth study based on methodology of Diewertje et al., 2016 [64]

^c^Only in a sub-set of participants (*n* = 60)

#### Sweetness preference and perceived taste intensity

Sweetness preference and perceived taste intensity are assessed at each study visit in six sweet test foods. Additionally, two control (salty) test foods are evaluated by participants to determine whether effects of sweetness exposure are specific for sweet taste. Since preference for sweetness strongly depends on the type of food [37, 38], and past experience (familiarity) with those foods, the design of the current study includes foods differing in food form (liquid, semi-solid and solid) and familiarity (familiar and unfamiliar). Table 5 gives an overview of the test foods together with their intensity levels. Each test food stimuli ranges from low sweet/salt to high sweet/salt, across five intensity levels (so-called L-2, L-1, L-0, L + 1, L + 2; adapted from Urbano et al. [65]). Test foods selected for the Sweet Tooth study were pre-tested over a period of 1 year, across four pilot studies, that included in total 127 participants ([66], full paper to be submitted).

**Table 5:** Test foods used in the preference and intensity testing with sweetness and saltiness concentration levels and percentages of added sweetener (sugar + non- and/or low-calorie sweeteners) and salt in % by weight, for each level. The stimuli are used to assess the primary (preference) and one of the secondary outcome measures in the Sweet Tooth trial

	**Test food**	**Food form**	**Serving size**	**Sweetener concentration**^a^ (% by weight)				
				**L-2**	**L-1**	**L-0**	**L + 1**	**L + 2**
Familiar	**Strawberry flavoured lemonade**^b^	Liquid	20 ml	0.00	1.26^c^	3.08^c^	8.56^c^	15.06^c^
	**Chocolate flavoured custard**^b^	Semi-Solid	15 g	3.41^c^	6.59^c^	12.37^c^	17.57^d^	26.33^d^
	**Plain Cake**^b^	Solid	20 g	9.13^c^	16.74^c^	19.15^d^	21.88^d^	25.10^d^
Unfamiliar	**Watermelon flavoured lemonade**	Liquid	20 ml	0.00	1.26^c^	3.08^c^	8.56^c^	15.06^c^
	**Elderflower flavoured custard**	Semi-Solid	15 g	3.61^c^	6.98^c^	13.05^c^	18.90^d^	27.59^d^
	**Tamarind flavoured cake**	Solid	20 g	9.13^c^	16.74^c^	19.15^c^	21.88^d^	25.10^d^
				**Salt concentration**^a^ **(% by weight)**				
				**L-2**	**L-1**	**L-0**	**L + 1**	**L + 2**
Familiar	**Gazpacho**	Liquid	20 ml	0.05	0.15	0.30	0.72	1.46
	**Butter cracker**	Solid	3.5 g	0.00	0.71	1.37	3.50	7.05

^a^Five sweetness/saltiness levels, with the middle level (L-0) representing the optimal sweetness/saltiness sensation, initially based on the quantity present in the commercial products or recipes as described by Urbano et al. [65], and to some degree adjusted after pilot testing with Dutch consumers

^b^Recipes for food preparation adapted from Urbano et al. [65]

^c^Added sucrose

^d^Added sucrose and liquid sweetener based on cyclamate and saccharin (Rio Zoetstof, Sweet Life AG, Switzerland)

The preferred sample is defined as the highest liking rating score, assessed with a rank-rating scale [67–70]. This, so called ranking on a scale method was selected based on the previously mentioned pilot studies. In short: participants are simultaneously presented with five food samples of the same food product, each sample corresponding to one level of the taste intensity range. They are then asked to taste and swallow a mouthful of each sample and rate it on liking using a single 100-unit Visual Analogue Scale (VAS). The used scale is end-anchored ‘like extremely’ and ‘dislike extremely’, and at the middle ‘neither like or dislike’. This scale also allows rating ties when two or more stimuli are equally liked.

Perceived taste intensity is recoded on a 100-unit VAS, end-anchored ‘not sweet/salty at all’ and ‘extremely sweet/salty’. Participants are asked to taste each stimulus and rate its sweetness or saltiness intensity. During taste intensity evaluation, stimuli are presented using a monadic protocol.

The order of presentation of the stimuli are randomised in both preference and taste intensity sessions. Stimuli are labelled with 3-digit random codes and provided in standardised volumes (see Table 5), at room temperature in translucent cups or served on small trays. Water is provided as a palate cleanser. Breaks of 30-60 s between stimuli tasting, are implemented to minimise possible carry-over effects. Both preference and perceived intensity testing take approximately 1 h. Testing takes place in eating behaviour booths of the Human Nutrition Research Unit of Wageningen University, under normal lighting and odour free conditions. Preference and perceived intensity ratings are recorded digitally using EyeQuestion Software (https://eyequestion.nl/). Both tests occur at every study visit, at standardised times.

#### Behavioural outcomes

Food choice and intake are measured during a breakfast buffet where a wide range of food products are served to participants ad libitum. Served breakfast foods mainly vary in taste modalities (sweet, savoury, neutral, bitter foods), to determine whether effects of sweetness exposure influence the intake of sweet foods. Participants are free to eat as much as they want and given 30 min. Food choice and intake are monitored covertly, and unconsumed foods returned to the kitchen are weighed. Proportions of sweet and non-sweet foods consumed are measured. These measurements also result in energy and macronutrient intake, which is calculated using the NEVO-codes [71] and nutrient calculation software programme Compl-Eat (www.compleat.nl, Wageningen University, Wageningen, NL) [72]. Additionally food choice is assessed at the end of the assessment visit, when participants are offered a take-away snack. Several snacks of different taste modalities (sweet, savoury and neutral) are offered and participants’ choice is recorded.

Sweet-liker type is assessed by a method proposed by Iatridi et al. [54]. With this method three distinct sweet-liker phenotypes can be distinguished: sweet liker (SL), sweet disliker (SD) and inverted U-shaped (IU) phenotype. Participants evaluate a sucrose solution of 1 mol/L sucrose on liking using a 100-unit VAS, and are classified as one of the three phenotypes, using the following cut off values SD: score ≤ 35; SL: score ≥ 65; IU: score 36–64 [54].

Food cravings are assessed using a translated version of The Control of Eating Questionnaire (CoEQ) [61]. The CoEQ consists of 21 questions designed to assess the type and intensity of food cravings, as well as subjective sensations regarding appetite and mood. It also includes two questions on desire to eat; one about desire to eat sweet foods and the other about desire to eat savoury foods. The CoEQ measures cravings across four components: craving control, positive mood, craving for sweet, and craving for savoury foods.

A modified version of the PrefQuest questionnaire [63] is used to assess taste preferences across four components related to liking for Sweet; Fat and Sweet; Salt; and Fat and Salt items. Firstly, the questionnaire was translated to Dutch by an professional translation bureau (in’to Languages, Wageningen, The Netherlands). Secondly, French foods, that are not typically eaten by the Dutch were identified, by two researchers and two dietitians, and excluded from the questionnaire. Thirdly, excluded French foods were exchanged by typically eaten Dutch foods that fall in the same taste and food category, using the Dutch taste database [1] and the Dutch Food Consumption survey 2012–2016 [73], respectively. For example, a Morteau sausage (a traditional French smoked sausage), which was in the Fat and Salt sensation and Meats food group [63], was exchanged for Rookworst (a traditional Dutch smoked sausage), classified under the Salt, Umami and Fat taste sensation group [1]. Additionally, images that did not follow the Dutch eating habits were replaced. For instance, we changed the type of bread to that most commonly eaten in the Netherlands.

Dietary taste patterns are assessed with a newly developed FFQ that assesses relative taste exposure through the measurement of specific food items based on frequency, amount and type of food consumed. The main purpose of the so-called Taste FFQ is to assess habitual dietary taste patterns in adults. The Dutch FFQTOOL™ was used to develop the Taste FFQ by selecting food items with the largest contributions to total intake and explained variance in energy intake (the Dutch Food Consumption survey 2012–2016 [73]), per taste cluster: Fat, Neutral, Sweet & Sour, Salt, Umami & Fat, Sweet & Fat and Bitter [1]. Additionally, to increase face validity and reliability, additional food items were added to the questionnaire, such as table salt and cinnamon. All in all, the Taste FFQ consists of 162 food items, including 7 Fat, 35 Neutral, 25 Sweet & Sour, 56 Salt Umami & Fat, 31 Sweet & Fat, and 8 Bitter tasting food items. The usability and applicability of taste FFQ was pre-tested in a sample of 52 participants and revised afterwards. Dietary data collected with the Taste FFQ allows the study of taste dietary patterns, where dietary patterns can be derived a priori (e.g., based on the intervention diet) and a posteriori (e.g., using principal component analysis, or cluster analysis). The newly developed Taste FFQ provides more insights on the dietary taste patterns in relation to the development of food preferences, weight and health status.

#### Anthropometric outcomes 

Anthropometric measurements are taken in light clothing without footwear using standard techniques. Height is measured with a stadiometer (SECA, Germany) to the nearest 0.1 cm. Weight is measured twice, using a calibrated digital weighing scale (SECA, Germany) to the nearest 0.1 kg. The average of the two measurements is included in the dataset. Waist and hip circumference are measured using a flexible tape (SECA 201, Germany) and recorded to the nearest 0.5 cm. Body composition (e.g. lean body mass, body fat percentage) is measured by a dual-energy X-ray absorptiometry (DEXA) scan (Lunar, United States).

#### Biochemical outcomes

At each study visit a fasting venous blood sample is obtained by a trained phlebotomist. Collected blood samples are centrifuged within two hours of collection and stored at -80 °C for later analysis. A broad range of biomarkers are measured to assess the effects of the intervention on diabetes and cardiovascular health, including fasting glucose, HbA1c, insulin, total cholesterol, high-density lipoprotein (HDL), low-density lipoprotein (LDL) and triglycerides. Total cholesterol, HDL, LDL, triglycerides, glucose and HbA1c are analysed in a clinical laboratory (Hospital Gelderse Vallei, Ede, the Netherlands), using the enzymatic methods (Atellica® CH analyzer) and/or High Performance Liquid Chromatography (HPLC). Insulin is measured with enzyme-linked immunosorbent assay (ELISA). Additionally, white blood cells are stored at -80 °C for future analysis to provide indicative effects of gene variants if deemed of interest [11, 12, 74, 75].

Glucose variability is assessed in a sub-group of 60 volunteers, over a 14-day period, at baseline, at the end of intervention and at the end of follow up period with the help of a commercially available glucose sensor (FreeStyle Libre Flash Glucose Monitoring System) [76, 77]. Participants wear the sensor on the back of their upper arm for up to 14 days and scan the sensor with a glucose reader every 6–8 h. This system automatically measures glucose levels in interstitial fluid every minute, and stores the readings in 15-min intervals. These data provide information on postprandial glucose responses and changes in glucose regulation.

#### Compliance measures

Adherence to diets is assessed with biomarkers of sweetener consumption in 24-h urine sample. Long-term sweetness exposure is confirmed at a group level, by measuring biomarker levels of: urinary sucrose and fructose [78–81] and urinary excretion of five commonly consumed LCSs: acesulfame-K, saccharin, sucralose, cyclamate and steviol glycosides [79, 82]. Adherence to the intervention is estimated by the mean group presence of urine biomarkers in gram or milligrams. Participants collect urine samples six times in total, having received written and verbal instructions on urine collection, three 100 mg para-aminobenzoid acid (PABA) tablets (KAL PABA, KALvitamines, Huizen, The Netherlands), together with urine containers containing preservative lithium dihydrogenphosphate. Urine collection starts after the first voiding after waking up and finishes after the first voiding after waking up the following day. Voiding times, the use of medications and/or nutritional supplements and possible deviations from the protocol (e.g., missing urine) are registered. Once collected, urine of each participant is mixed, weighed, aliquoted and stored at − 80 °C until further analyses. Completeness of urine sampling is checked by the recovery dosage of PABA, using 78% of ingested PABA as an acceptable recovery rate [83]. Urinary fructose and sucrose [78], LCSs [82], sodium and nitrogen levels are measured in urine samples as biomarkers for daily intakes of sucrose, LCSs, sodium and protein intake, respectively.

Moreover, compliance is assessed using 24-h dietary recalls. Adherence to the intervention is estimated as exposure to different taste modalities (e.g. sweet tasting foods) as % energy consumed, as % weight consumed or frequency consumed. Web-based 24-h recalls are completed using the software programme Compl-Eat (www.compleat.nl) [72]. Participants fill out this web-based 24-h recall nine times; once at baseline, once every month during the intervention and twice in the follow up period. From this dietary data an average daily percent of energy from sweet tasting food is calculated. Additionally, average daily energy consumed from macronutrients and micronutrient intakes is estimated using the most recent Dutch food composition table [73].

#### Intervention moderators

The general inclusion questionnaire includes questions about; age; sex; education level; current work situation; health and history of diseases such as diabetes mellitus, cardiovascular health, taste or smell difficulties, allergies, general disorders and medication use; living environment and social activities such as diet, smoking status, nutritional supplements, alcohol and drug usage. Habitual physical activity is assessed to explore a potential moderating effect on sweetness preferences and other outcomes, using a previously validated Short Questionnaire to Assess Health-enhancing physical activity (SQUASH) [62]. The questionnaire measures the frequency (days per week), duration (hour and minutes) and intensity (low, moderate, high) of different physical activities referring to a normal week in recent months. Physical activities include occupation, leisure time, household, transportation means and other daily activities [62]. Blinding to the study aims and satisfaction with the diet are assessed with a study debrief questionnaire, at the end of the last assessment visit. Debrief questionnaire is an open-ended questionnaire that aims to gather participants' opinions after the end of intervention, and to cross check the cover story.

#### Adverse events

Adverse events will be recorded in the case report form and are informed by a medical investigator to the Medical Ethical Committee. Although we do not intend to formally analyze adverse events, data will be included in future publications. Although the HSE group is exposed to more LCSs, compared to LSE and RSE groups, we do not anticipate any adverse events to occur (e.g. flatulence, bloating, or abdominal discomfort). The provided commercially available products only contain small amounts of LCSs, that are considerably lower than accepted daily intakes. However, any alteration to one’s diet may result in occurrence of adverse events, yet this risk is low and present with any dietary intervention.

### Outcome assessment procedure

**Fig. 2:**
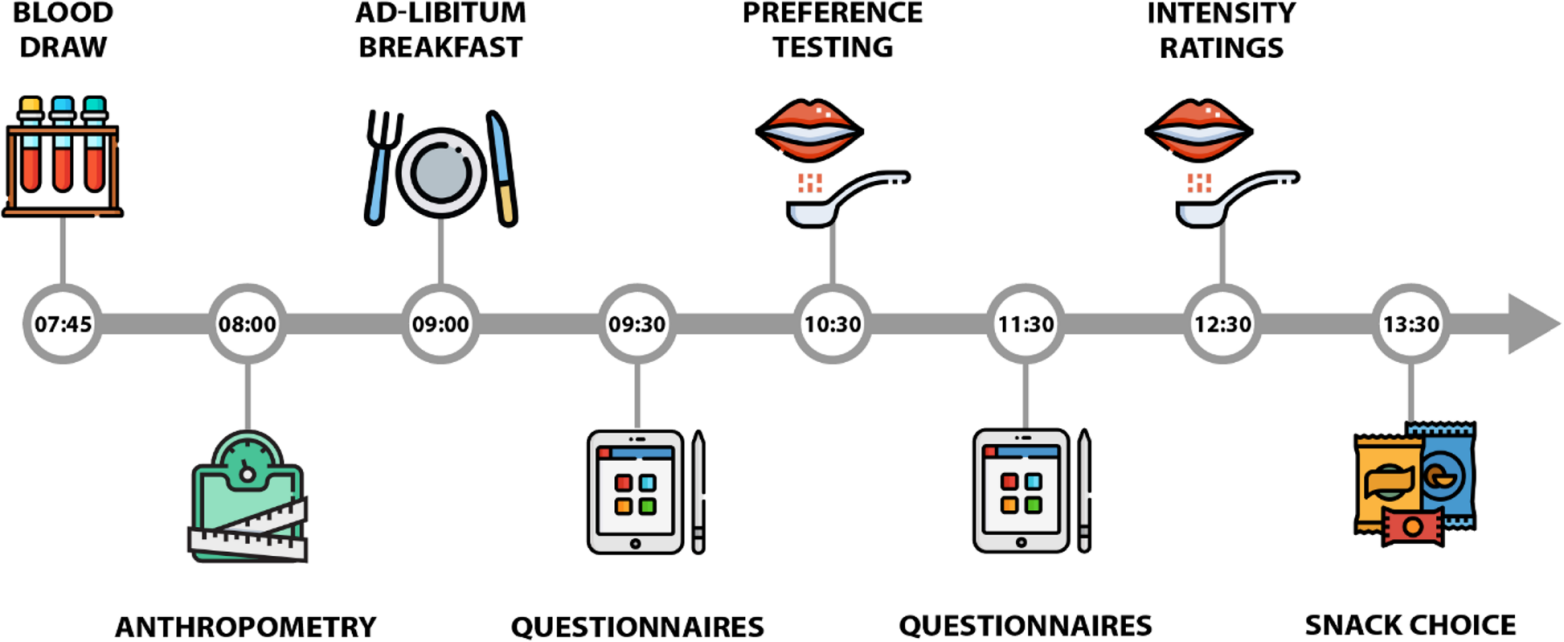
Assessment visit timeline overview lasting approx. 6 h. Measurements taken at the assessment visit, in chronological order, are; blood biomarkers related to CVD and diabetes, body weight, hip-waist circumference, body composition, food choice and intake (ad libitum test breakfast meal), dietary taste preferences (PrefQuest), food cravings (Control of Eating questionnaire), sweet-liker status, sweet and salt taste preferences (sensory evaluation), dietary taste patterns (Taste FFQ), physical activity level (SQUASH questionnaire), sweet and salt taste intensity perception (sensory test) and food choice (snack choice)

Study assessments are conducted at baseline, 1, 3, 6, 7 and 10 months at the Human Nutrition Research Facilities, located on the Wageningen University Campus, the Netherlands. Each assessment visit follows a strict standardised operating procedure and lasts approximately 6 h. Figure 2 shows the assessment visit timeline with assessments in chronological order.

### Statistical analyses plan

Study data will be handled according to the General Data Protection Regulation Act (EU GDPR), and will be anonymised and archived in password protected servers. Prior to data analysis, normality of the data will be tested. Non-normally distributed data will be transformed or analysed using non-parametric tests, if deemed necessary. Statistical significance will be set at *p* < 0.05. Descriptive statistics will be provided for each of the three intervention groups at baseline and will include demographic, dietary and lifestyle information. Continuous data will be summarized using means, SD and 95% confidence intervals, while categorical variables will be summarised using counts and percentages.

First, to determine whether there is a shift in sweetness preference between baseline and month 6, statistical techniques appropriate for longitudinal data analysis, linear mixed effects models, will be used, with treatment (LSE, RSE and HSE), time (baseline and 6-months) and treatment x time as fixed factors and participant number as a random factor. Significant effects will be further investigated by Posthoc analyses adjusted for multiple testing. Our main interest is in the change in preference scores between any two intervention groups. Second, similar linear mixed effects models will be used to explore effects of sweet taste exposure on the other dependent measures over time. In light of the research in this area, we will explore differences between intervention groups and secondary outcome variables (pre-registered), and explore related but separate questions of whether glucose variability, body composition, food cravings, blood biomarkers are related to sweetness exposure, age, gender, sweet liker status and BMI. By comparing group means of urine extraction of urinary sucrose, fructose, and LCSs between intervention groups, the level of compliance will be evaluated. Linear mixed effects models will be adjusted for covariates where appropriate and both unadjusted and adjusted models will be reported. If main effects on a dependent variable are identified, Bonferroni post hoc tests will be applied to identify statistically significant differences between means. The difference in means will provide an effect of the intervention on the outcomes. Analysis will be conducted on both an intention-to-treat (ITT) and a per-protocol basis. The ITT analysis will be the primary analysis. The per-protocol analysis, however, will help us determine whether the effects are the result of individuals adhering to the procedure and consuming the provided intervention foods. In the event of missing data due to drop-out, the outcome variables that have not been recorded will be treated as missing data. Unblinding will occur at the conclusion of the study, to determine the effect of the intervention.

## Discussion

This paper describes the protocol for the Sweet Tooth trial to explore the effect of a 6-month low, regular and high dietary sweetness exposure on sweet food and beverage preferences. The intervention has been designed using recently developed methodology enabling the assessment of sweetness exposure within a diet, and the study design includes a number of novel methodologies. To our knowledge, this is the first trial to study the effect of long-term low, regular and high sweetness exposure, where exposure to sweet taste comes from various sources and sweetness of the whole diet is taken into account. Furthermore, preference for sweetness is assessed with various test foods, across a wide range of intensity levels, varying in form (liquids, semi-solids and solids) and familiarity. Preference is assessed with ranking on a scale; this methodology combines the sensitivity of ranking from side‐by‐side assessment of tested samples, coupled with the benefits of rating by providing indication of spacing between tested samples [70]. The current study also includes several dietary assessment methods, including web-based 24-h recalls, Taste FFQ and biochemical markers (including urinary recovery of sugars and LCSs). The dietary assessment approaches used in the Sweet Tooth study will help us gain a comprehensive picture of sweetness exposure and compliance. This is important since it will ensure the validity of results [84]. As such, this work has the potential to expand the current understanding of how long-term sweetness exposure influences sweetness preferences, while also providing information about resilience of sweetness preferences, in terms of effect size, direction and duration. In addition, the effect on anthropometric biochemical outcomes is assessed, thus potentially advancing our current understanding of the impact of sweetness exposure on body weight/composition and other health related outcomes.

One of the main challenges of this comprehensive trial is the recruitment [85]. The recruitment strategy involves a variety of methods to attract a heterogenous group of volunteers. Once recruited we try to minimise the time between obtaining consent and participation. Another important challenge with these long-term dietary interventions is keeping participants motivated and compliant [85]. The Sweet Tooth study is a randomized controlled trial with partial food provision, which means that 50% of the food items are provided, while the other 50% has to be arranged by participants themselves. To maximise compliance, participants are provided with these intervention foods on a biweekly basis and receive a monthly dietary booklet which includes daily menus, tips (e.g. what to choose when in a restaurant) and food checklists, to guide them through the intervention. Furthermore, participants are provided with a wide range of different food products and brands, to give them some freedom over their allocated diet and they have regular appointments with the dietitian. In addition, positive experiences such as meeting new people, trying new foods, visiting research facilities are being highlighted and encouraged. Another potential limitation of this study is participant dropout. Interventions lasting over several months can be linked to higher dropout rates [84]. To account for this, we plan to enrol 20% more participants than required based on sample size calculation. Moreover, to minimize drop-out rates, no run-in period had been implemented in the study design. Incorporating a run-in period would likely increase the time burden, in turn potentially leading to non-compliance or increased dropout rates. When looking at the impact of sweet taste exposure on the outcomes of the trial, we will not be able to determine whether the effects are due to LCSs or sugars because all intervention groups are given foods that are both sweetened with sugar and foods that are sweetened with LCSs. The use of sugar and LCS in all groups and in differing amounts was considered critical in the study design to ensure the study was an intervention of ‘sweet foods exposure’, rather than ‘a intervention of sugar’ or ‘an intervention of LCS’ consumption. More importantly, the use of LCSs makes it possible to standardize the amount energy from carbohydrates across all three intervention groups. It also should be noted that this study uses assessment tools that have not yet been validated, such as the Taste FFQ. The Taste FFQ was developed to assess dietary taste exposure, and will be assessed for validity as part of this study, against 24-h recalls and biochemical markers. The primary outcome for the study, preference for sweetness, is a subjective perception, that can only be assessed using subjective measurements. The methods we are using have been carefully developed [66], based on established techniques and published papers [65]. This also holds for some of our other outcomes, such as assessments of taste intensity and sweet-liker status. The methods we use to measure these perceptions have also been validated [55, 86]. Objective measurements for subjective perceptions are not available due to their subjective nature, although the use of proxy measures may be possible.

It also has to be noted that the Sweet Tooth trial is being conducted during the Covid-19 pandemic. Therefore, pandemic-related restrictions, such as lockdowns, are likely to influence the trial progress. For example, as a result of restricted access to research facilities and social distancing measures, a delay in data collection is expected. In addition, local restrictions might influence the dietary patterns and eating behaviours of participants. However, this is unlikely, since the intervention and delivery of provided foods will remain unchanged. Any related changes will also impact all participants in all dietary intervention groups equally. We speculate that the reduced social interaction and traveling restrictions might even increase dietary compliance.

## Conclusion

This 6-month randomized controlled trial with partial food provision will add to the evidence on sweetness exposure and its effects on sweetness preferences, eating behaviour, body weight and health related outcomes. Findings from this study will generate data required to support evidence-based recommendations for dietary guidelines.

### Trial status

METC approval: NL72134.081.19. Approval obtained on July 28^th^, 2020, recruitment began in September 2020, and study measuring from October 2020 onwards. Estimated recruitment end is September 2023.

## Figures and Tables

**Fig. 3:**
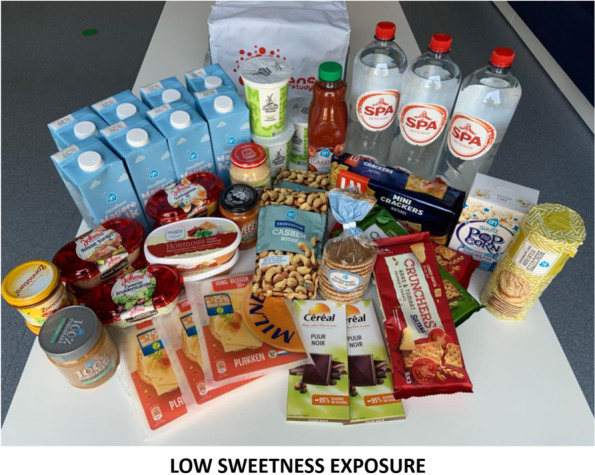
Provided intervention foods delivered on a biweekly basis including solid, semi-solid and liquid foods for low sweetness exposure group

**Fig. 4:**
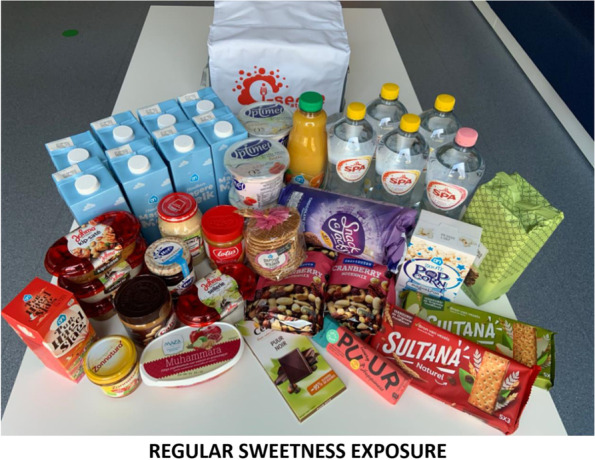
Provided intervention foods delivered on a biweekly basis including solid, semi-solid and liquid foods for regular sweetness exposure group

**Fig. 5:**
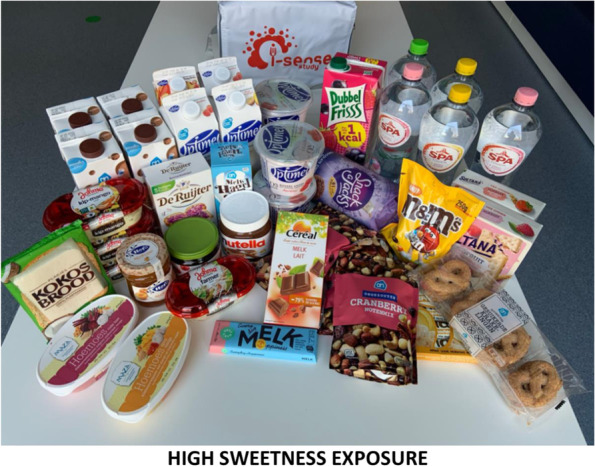
Provided intervention foods delivered on a biweekly basis including solid, semi-solid and liquid foods for high sweetness exposure group

## Data Availability

Data are owned by Wageningen University (NL). Processed data will be shared with Bournemouth University (UK) and anonymised dataset will be available immediately following the publication. No end date.

## Appendix 1

Tables 6, 7 and 8

**Table 6:** Daily menu example for the Low-sweet Exposure Group with highlighted and in bold provided intervention foods

	**Food product**	**Item brand/type**
**Breakfast**	Bread	Any
	Margarine	Low fat margarine 40% fat
	**Vegetable spread**	**Mister Kitchen vegetable spread**
	**Egg salad**	**Johma Eisalade**
	**Cheese**	**Milner Belegen kaas 30 + **
	**Skimmed milk**	**AH Magere melk**
**Snack**	Coffee/tea/water	Any
	Fruit	Any
	**Savoury biscuits**	**Sultana Crunchers**
**Lunch**	Bread	Any
	Margarine	Low fat margarine 40% fat
	**Vegetable spread**	**Heunz Dandwich spread naturel**
	**Pepper spread**	**Zonnatura Paprika spread**
	Meat	Any
	**Skimmed milk**	**AH Magere melk**
**Snack**	Coffee/tea/water	Any
	Fruit	Any
	**Unsalted cashew nuts**	**AH Cashewnoten ongezouten**
**Dinner**	Spaghetti Bolognese	
	Water	
**Snack**	Coffee/tea/water	Any
	**Sparkling water**	**Spa Intense bruisend mineraalwater**
	**Neutral mini crackers**	**LU Mini crackers naturel**

**Table 7:** Daily menu example for the Regular-sweet Exposure Group with highlighted and in bold provided intervention foods

	**Food product**	**Item brand/type**
**Breakfast**	Bread	Any
	Low fat margarine 40% fat	Low fat margarine 40% fat
	**Peanut butter**	**Calve Pindakaas**
	**Fruit Jam with less sugar**	**Hero Minder zoet jam**
	Kaas	Any
	**Skimmed milk**	**AH Magere melk**
**Snack**	Coffee/tea/water	Any
	Fruit	Any
	**Fruit biscuits**	**Sultana Fruitbiscuit**
**Lunch**	Bread	Any
	Margerine	Low fat margarine 40% fat
	**Celery salad**	**Joma Selleriesalade**
	**Pepper spread**	**Zonnatura Paprika spread**
	Meat	Any
	**Skimmed milk**	**AH Magere melk**
**Snack**	Coffee/tea/water	Any
	Fruit	Any
	**Unsalted nuts with cranberries**	**AH Cranberrymix ongezouten**
**Dinner**	Spaghetti Bolognese	
	Water	
**Snack**	Coffee/tea/water	Any
	**Flavoured water**	**Spa Touch bruisend**
	**Rice cracker with chocolate flavour**	**Rijstwafels met chocolade**

**Table 8:** Daily menu example for the High-sweet Exposure Group with highlighted provided intervention foods

	**Food product**	**Item brand/type**
**Breakfast**	Bread	Any
	Margarine	Low fat margarine 40% fat
	**Peanut butter with coconut and maple syrup**	**Mister Kitchen’s Pindakkas kokos maple**
	**Fruit jam**	**Hero fruit jam**
	**Chocolate milk**	**AH Choc drink**
**Snack**	Coffee/tea/water	Any
	Fruit	Any
	**Fruit and yogurt biscuits**	**Sultana YoFruit**
**Lunch**	Bread	Any
	Margarine	Low fat margarine 40% fat
	**Chocolate spread**	**Nutella**
	**Apple syrup**	**AH Biologisch Appelstroop**
	**Yogurt drink**	**Optimel Drinkyoghurt 0% vet**
**Snack**	Coffee/tea/water	Any
	Fruit	Any
	**Unsalted nuts with cranberries**	**AH Cranberrymix ongezouten**
**Dinner**	Spaghetti Bolognese	
	Water	
**Snack**	Coffee/tea/water	Any
	**Flavoured water**	**Spa Touch bruisend**
	**Rice cracker with chocolate flavour**	**Rijstwafels met chocolade**

## Appendix 2

Figures 3, 4 and 5

## Abbreviations

BMI: Body mass index
CoEQ: The Control of Eating Questionnaire
DEXA: Dual-energy X-ray absorptiometry
DNFCS: Dutch Food Consumption Survey
ELISA: Enzyme-linked immunosorbent assay
FFQ: Food Frequency questionnaire
HDL: High-density lipoprotein
HSE: High Sweetness Exposure
IU: Inverted U-shaped phenotype
ITT: Intention-to-treat
LDL: Low-density lipoprotein
LCSs: Low-calorie sweeteners
LSE: Low Sweetness Exposure
PABA: Para-aminobenzoid acid
RSE: Regular Sweetness Exposure
SD: Sweet Disliker phenotype
SL: Sweet Liker phenotype
SQUASH: The Short Questionnaire to Assess Health-enhancing physical activity
VAS: Visual analogue scale
WHO: World Health Organization

## References

[1] van Langeveld A, Teo PS, de Vries JHM, Feskens EJM, de Graaf C, Mars M (2018). Dietary taste patterns by sex and weight status in the Netherlands. Br J Nutr.

[2] Wittekind A, Higgins K, McGale L, Schwartz C, Stamataki NS, Beauchamp GK, et al. A workshop on “Dietary Sweetness-Is It an Issue?” Int J Obes (Lond). 2018;42(4):934–8. 10.1038/ijo.2017.296 PMC5984094 29211705

[3] Wittekind A, Walton J (2014). Worldwide trends in dietary sugars intake. Nutr Res Rev.

[4] ODPHP OoDPaHP. Dietary Guidelines for Americans: U.S. Department of Health and Human Services and U.S. Department of Agriculture; 2015 [Available from: https://health.gov/dietaryguidelines/2015/guidelines/.

[5] Guideline: Sugars Intake for Adults and Children. Geneva: World Health Organization; 2015. Available from: https://www.ncbi.nlm.nih.gov/books/NBK285537/. 25905159

[6] England PH. Sugar reduction: the evidence for action. Annexe 5: food supply.: Public Health England; 2015. Available from: https://assets.publishing.service.gov.uk/government/uploads/system/uploads/attachment_data/file/470176/Annexe_5._Food_Supply.pdf. [Cited 26 Feb 2021].

[7] Organization PAH. Pan American Health Organization nutrient profile model.: Pan American Health Organization. ; 2016. Available from: https://iris.paho.org/bitstream/handle/10665.2/18621/9789275118733_eng.pdf?sequence=9& isAllowed=y. [Cited 26 Feb 2021].

[8] Union E. Framework for national initiatives on selected nutrients. Annex II: added sugars. 2011. Available from: https://ec.europa.eu/health/sites/health/files/nutrition_physical_activity/docs/added_sugars_en.pdf. [Cited 26 Feb 2021].

[9] Venditti C, Musa-Veloso K, Lee HY, Poon T, Mak A, Darch M (2020). Determinants of Sweetness Preference: A Scoping Review of Human Studies. Nutrients.

[10] Steiner JE, Glaser D, Hawilo ME, Berridge KC (2001). Comparative expression of hedonic impact: affective reactions to taste by human infants and other primates. Neurosci Biobehav Rev.

[11] Hwang LD, Lin C, Gharahkhani P, Cuellar-Partida G, Ong JS, An J, Gordon SD, Zhu G, MacGregor S, Lawlor DA, Breslin PAS, Wright MJ, Martin NG, Reed DR. New insight into human sweet taste: a genome-wide association study of the perception and intake of sweet substances. Am J Clin Nutr. 2019;109(6):1724–37. 10.1093/ajcn/nqz043. 10.1093/ajcn/nqz043 PMC6537940 31005972

[12] Keskitalo K, Knaapila A, Kallela M, Palotie A, Wessman M, Sammalisto S (2007). Sweet taste preferences are partly genetically determined: identification of a trait locus on chromosome 16. Am J Clin Nutr.

[13] Bertino M, Beauchamp GK, Engelman K (1982). Long-term reduction in dietary-sodium alters the taste of salt. Am J Clin Nutr.

[14] Bertino M, Beauchamp GK, Engelman K (1986). Increasing dietary salt alters salt taste preference. Physiol Behav.

[15] Wise PM, Nattress L, Flammer LJ, Beauchamp GK (2016). Reduced dietary intake of simple sugars alters perceived sweet taste intensity but not perceived pleasantness. Am J Clin Nutr.

[16] Appleton KM, Tuorila H, Bertenshaw EJ, de Graaf C, Mela DJ (2018). Sweet taste exposure and the subsequent acceptance and preference for sweet taste in the diet: systematic review of the published literature. Am J Clin Nutr.

[17] Breslin PAS, Izumi A, Tharp A, Ohkuri T, Yokoo Y, Flammer LJ (2021). Evidence that human oral glucose detection involves a sweet taste pathway and a glucose transporter pathway. PLoS ONE.

[18] O’Brien P, Han G, Ganpathy P, Pitre S, Zhang Y, Ryan J, et al. Chronic Effects of a High Sucrose Diet on Murine Gastrointestinal Nutrient Sensor Gene and Protein Expression Levels and Lipid Metabolism. Int J Mol Sci. 2020;22(1):137. 10.3390/ijms22010137 PMC7794826 33375525

[19] Mattes RD, Popkin BM (2009). Nonnutritive sweetener consumption in humans: effects on appetite and food intake and their putative mechanisms. Am J Clin Nutr.

[20] Gilhooly CH, Das SK, Golden JK, McCrory MA, Dallal GE, Saltzman E (2007). Food cravings and energy regulation: the characteristics of craved foods and their relationship with eating behaviors and weight change during 6 months of dietary energy restriction. Int J Obes (Lond).

[21] Martin CK, Rosenbaum D, Han H, Geiselman PJ, Wyatt HR, Hill JO (2011). Change in food cravings, food preferences, and appetite during a low-carbohydrate and low-fat diet. Obesity (Silver Spring).

[22] Chao A, Grilo CM, White MA, Sinha R (2014). Food cravings, food intake, and weight status in a community-based sample. Eat Behav.

[23] Martin CK, O’Neil PM, Tollefson G, Greenway FL, White MA. The association between food cravings and consumption of specific foods in a laboratory taste test. Appetite. 2008;51(2):324–6. 10.1016/j.appet.2008.03.002 PMC2748323 18417253

[24] Valentin D, Chrea C, Nguyen DH. Taste–odour interactions in sweet taste perception. In Optimising sweet taste in foods. Cambridge: Woodhead Publishing; 2006. pp. 66–84. 10.1533/9781845691646.1.66.

[25] Hedrick VE, Davy BM, You W, Porter KJ, Estabrooks PA, Zoellner JM (2017). Dietary quality changes in response to a sugar-sweetened beverage-reduction intervention: results from the Talking Health randomized controlled clinical trial. Am J Clin Nutr.

[26] Piernas C, Tate DF, Wang XS, Popkin BM (2013). Does diet-beverage intake affect dietary consumption patterns? Results from the Choose Healthy Options Consciously Everyday (CHOICE) randomized clinical trial. Am J Clin Nutr.

[27] Tey SL, Brom RC, Gray AR, Chisholm AW, Delahunty CM (2012). Long-term consumption of high energy-dense snack foods on sensory-specific satiety and intake. Am J Clin Nutr.

[28] Ebbeling CB, Feldman HA, Steltz SK, Quinn NL, Robinson LM, Ludwig DS (2020). Effects of Sugar-Sweetened, Artificially Sweetened, and Unsweetened Beverages on Cardiometabolic Risk Factors, Body Composition, and Sweet Taste Preference: A Randomized Controlled Trial. J Am Heart Assoc.

[29] Griffioen-Roose S, Hogenkamp PS, Mars M, Finlayson G, de Graaf C (2012). Taste of a 24-h diet and its effect on subsequent food preferences and satiety. Appetite.

[30] Asao K, Rothberg AE, Arcori L, Kaur M, Fowler CE, Herman WH (2016). Sweet taste preferences before and after an intensive medical weight loss intervention. Obes Sci Pract.

[31] Desor JA, Beauchamp GK (1987). Longitudinal changes in sweet preferences in humans. Physiol Behav.

[32] Goodman EL, Breithaupt L, Watson HJ, Peat CM, Baker JH, Bulik CM (2018). Sweet taste preference in binge-eating disorder: A preliminary investigation. Eat Behav.

[33] Kleifield EI, Lowe MR (1991). Weight loss and sweetness preferences: The effects of recent versus past weight loss. Physiol Behav.

[34] Pomerleau CS, Garcia AW, Drewnowski A, Pomerleau OF (1991). Sweet taste preference in women smokers: Comparison with nonsmokers and effects of menstrual phase and nicotine abstinence. Pharmacol Biochem Behav.

[35] Szczygiel EJ, Cho S, Snyder MK, Tucker RM (2019). Associations between chemosensory function, sweet taste preference, and the previous night’s sleep in non-obese males. Food Qual Prefer.

[36] Zandstra EH, de Graaf C, van Trijp HCM, van Staveren WA (1999). Laboratory hedonic ratings as predictors of consumption. Food Qual Prefer.

[37] Divert C, Chabanet C, Schoumacker R, Martin C, Lange C, Issanchou S (2017). Relation between sweet food consumption and liking for sweet taste in French children. Food Qual Prefer.

[38] Abdallah L, Chabert M, Le Roux B, Louis-Sylvestre J (1998). Is pleasantness of biscuits and cakes related to their actual or to their perceived sugar and fat contents?. Appetite.

[39] Te Morenga L, Mallard S, Mann J (2012). Dietary sugars and body weight: systematic review and meta-analyses of randomised controlled trials and cohort studies. BMJ.

[40] Gibson S (2008). Sugar-sweetened soft drinks and obesity: a systematic review of the evidence from observational studies and interventions. Nutr Res Rev.

[41] Lim S, Zoellner JM, Lee JM, Burt BA, Sandretto AM, Sohn W (2009). Obesity and sugar-sweetened beverages in African-American preschool children: a longitudinal study. Obesity (Silver Spring).

[42] Bobowski N, Mennella JA (2017). Personal Variation in Preference for Sweetness: Effects of Age and Obesity. Child Obes.

[43] Cox DN, Hendrie GA, Carty D (2016). Sensitivity, hedonics and preferences for basic tastes and fat amongst adults and children of differing weight status: A comprehensive review. Food Qual Prefer.

[44] Ettinger L, Duizer L, Caldwell T (2012). Body fat, sweetness sensitivity, and preference: determining the relationship. Can J Diet Pract Res.

[45] Pepino MY, Mennella JA (2012). Habituation to the pleasure elicited by sweetness in lean and obese women. Appetite.

[46] Beauchamp GK (2016). Why do we like sweet taste: A bitter tale?. Physiol Behav.

[47] Deglaire A, Mejean C, Castetbon K, Kesse-Guyot E, Hercberg S, Schlich P (2015). Associations between weight status and liking scores for sweet, salt and fat according to the gender in adults (The Nutrinet-Sante study). Eur J Clin Nutr.

[48] van Langeveld AWB, Teo PS, de Vries JHM, Feskens EJM, de Graaf C, Mars M (2018). Dietary taste patterns by sex and weight status in the Netherlands. Br J Nutr.

[49] Lampure A, Castetbon K, Deglaire A, Schlich P, Peneau S, Hercberg S (2016). Associations between liking for fat, sweet or salt and obesity risk in French adults: a prospective cohort study. Int J Behav Nutr Phys Act.

[50] World Medical A (2001). World Medical Association Declaration of Helsinki Ethical principles for medical research involving human subjects. Bull World Health Organ.

[51] Rogers PJ, Appleton KM (2021). The effects of low-calorie sweeteners on energy intake and body weight: a systematic review and meta-analyses of sustained intervention studies. Int J Obes (Lond).

[52] Liem DG, de Graaf C (2004). Sweet and sour preferences in young children and adults: role of repeated exposure. Physiol Behav.

[53] Mueller C, Kallert S, Renner B, Stiassny K, Temmel AF, Hummel T (2003). Quantitative assessment of gustatory function in a clinical context using impregnated taste trips. Rhinology.

[54] Iatridi V, Hayes JE, Yeomans MR (2019). Quantifying Sweet Taste Liker Phenotypes: Time for Some Consistency in the Classification Criteria. Nutrients.

[55] Iatridi V, Hayes JE, Yeomans MR (2019). Reconsidering the classification of sweet taste liker phenotypes: A methodological review. Food Qual Prefer.

[56] Altman DG, Bland JM (1999). How to randomise. BMJ.

[57] Gezondheidsraad. Aanbieding advies Richtlijnen goede voeding 2015. In: Netherlands HCot, editor. The Hague: Health Council of the Netherlands; 2015.

[58] Bolhuis DP, Lakemond CMM, de Wijk RA, Luning PA, de Graaf C. Both a higher number of sips and a longer oral transit time reduce ad libitum intake. Food Quality and Preference. 2014;32(Part C):234–40.

[59] Viskaal-van Dongen M, Kok FJ, de Graaf C (2011). Eating rate of commonly consumed foods promotes food and energy intake. Appetite.

[60] van Langeveld AWB. Matters of taste: Dietary taste patterns in the Netherlands: Wageningen University; 2018.

[61] Dalton M, Finlayson G, Hill A, Blundell J (2015). Preliminary validation and principal components analysis of the Control of Eating Questionnaire (CoEQ) for the experience of food craving. Eur J Clin Nutr.

[62] Wendel-Vos G (2003). Reproducibility and relative validity of the short questionnaire to assess health-enhancing physical activity. J Clin Epidemiol.

[63] Deglaire A, Méjean C, Castetbon K, Kesse-Guyot E, Urbano C, Hercberg S (2012). Development of a questionnaire to assay recalled liking for salt, sweet and fat. Food Qual Prefer.

[64] Sluik D, Geelen A, de Vries JHM, Eussen SJPM, Brants HAM, Meijboom S (2016). A national FFQ for the Netherlands (the FFQ-NL 1.0): validation of a comprehensive FFQ for adults. Br J Nutr.

[65] Urbano C, Deglaire A, Cartier-Lange E, Herbreteau V, Cordelle S, Schlich P (2016). Development of a sensory tool to assess overall liking for the fatty, salty and sweet sensations. Food Qual Prefer.

[66] Cad EM, Tang CS, Mars M, Appleton KM, de Graaf K. When is it too sweet? Measuring sweetness preferences in foods amongst Dutch consumers. Appetite. 2022;169:105533. ISSN 0195-6663. 10.1016/j.appet.2021.105533.

[67] Kim K-O, O’Mahony M. A New Approach to Category Scales of Intensity I: Traditional Versus Rank-Rating. J Sens Stud. 1998;13(3):241–9.

[68] Sung Y-T, Wu J-S (2018). The Visual Analogue Scale for Rating, Ranking and Paired-Comparison (VAS-RRP): A new technique for psychological measurement. Behav Res Methods.

[69] Overview of applicable sensory evaluation techniques, Editor(s): Hildegarde Heymann, Susan E. Ebeler. Sensory and Instrumental Evaluation of Alcoholic Beverages. Academic Press; 2017. p. 34-71. ISBN 9780128027271. 10.1016/B978-0-12-802727-1.00003-X.

[70] Kemp SE, Hort J, Hollowood T. Descriptive Analysis in Sensory Evaluation2018.

[71] Milieu RRvVe. NEVO-online versie 2019/6.0 Bilthoven, Nederland2019 [Available from: https://nevo-online.rivm.nl/.

[72] Meijboom S, van Houts-Streppel MT, Perenboom C, Siebelink E, van de Wiel AM, Geelen A (2017). Evaluation of dietary intake assessed by the Dutch self-administered web-based dietary 24-h recall tool (Compl-eat) against interviewer-administered telephone-based 24-h recalls. J Nutr Sci.

[73] van Rossum C, Nelis K, Wilson C, Ocké M. National dietary survey in 2012–2016 on the general population aged 1–79 years in the Netherlands. EFSA Support Publ. 2018;15(9):1488E. 10.2903/sp.efsa.2018.EN-1488.

[74] Bachmanov AA, Bosak NP, Floriano WB, Inoue M, Li X, Lin C (2011). Genetics of sweet taste preferences. Flavour Fragr J.

[75] Park S, Liu M, Song MY (2020). Mental stress and physical activity interact with the genetic risk scores of the genetic variants related to sweetness preference in high sucrose-containing food and glucose tolerance. Food Sci Nutr.

[76] Bailey T, Bode BW, Christiansen MP, Klaff LJ, Alva S (2015). The Performance and Usability of a Factory-Calibrated Flash Glucose Monitoring System. Diabetes Technol Ther.

[77] Freestyle BA, System LGM (2018). Clin Diabetes.

[78] Abreu TC, Hulshof PJM, Boshuizen HC, Trijsburg L, Gray N, de Vries JHM (2021). Validity Coefficient of Repeated Measurements of Urinary Marker of Sugar Intake Is Comparable to Urinary Nitrogen as Marker of Protein Intake in Free-living Subjects. Cancer Epidemiol Biomarkers Prev.

[79] Gallagher AM, Logue C. Biomarker approaches to assessing intakes and health impacts of sweeteners: challenges and opportunities. Proc Nutr Soc. 2019;78(3):463–72. 10.1017/S0029665119000594. 10.1017/S0029665119000594 31023397

[80] Tasevska N (2015). Urinary Sugars–A Biomarker of Total Sugars Intake. Nutrients.

[81] Tasevska N, Midthune D, Tinker LF, Potischman N, Lampe JW, Neuhouser ML (2014). Use of a urinary sugars biomarker to assess measurement error in self-reported sugars intake in the nutrition and physical activity assessment study (NPAAS). Cancer Epidemiol Biomarkers Prev.

[82] Logue C, Dowey LRC, Verhagen H, Strain JJ, O’Mahony M, Kapsokefalou M, et al. A Novel Urinary Biomarker Approach Reveals Widespread Exposure to Multiple Low-Calorie Sweeteners in Adults. J Nutr. 2020;150(9):2435–41. 10.1093/jn/nxaa184 32678445

[83] Jakobsen J, Pedersen AN, Ovesen L (2003). Para-aminobenzoic acid (PABA) used as a marker for completeness of 24 hour urine: effects of age and dosage scheduling. Eur J Clin Nutr.

[84] Lucey A, Heneghan C, Kiely ME (2016). Guidance for the design and implementation of human dietary intervention studies for health claim submissions. Nutr Bull.

[85] Crichton GE, Howe PR, Buckley JD, Coates AM, Murphy KJ, Bryan J (2012). Long-term dietary intervention trials: critical issues and challenges. Trials.

[86] Armitage RM, Iatridi V, Yeomans MR (2021). Understanding sweet-liking phenotypes and their implications for obesity: Narrative review and future directions. Physiol Behav.

